# Could clinical photochemical internalisation be optimised to avoid neuronal toxicity?

**DOI:** 10.1016/j.ijpharm.2017.05.071

**Published:** 2017-08-07

**Authors:** Caitriona O’Rourke, Colin Hopper, Alexander J. MacRobert, James B. Phillips, Josephine H. Woodhams

**Affiliations:** a Division of Surgery & Interventional Science, University College London, London, UK; b Department of Biomaterials & Tissue Engineering, UCL Eastman Dental Institute, University College London, London, UK; c Academic Unit of Oral and Maxillofacial Surgery, UCL Eastman Dental Institute, London, UK

**Keywords:** Tetraphenylchlorin disulfonate (PubChem CID: 44177671), Meso-tetraphenylporphine (PubChem CID: 70186), Bleomycin (PubChem CID: 5360373), PCI, Photochemical Internalisation, PDT, Photodynamic therapy, ROS, Reactive oxygen species, HNC, Head and neck cancer, TPPS_2a_, Meso-tetraphenylporphine, TPCS_2a_, Tetraphenylchlorin disulfonate, 3D, Three-dimensional, DRG, Dorsal root ganglion, CNS, Central nervous system, Photochemical Internalisation, Nervous system, 3D culture models, Bleomycin, Photosensitisers

## Abstract

Photochemical Internalisation (PCI) is a novel drug delivery technology in which low dose photodynamic therapy (PDT) can selectively rupture endo/lysosomes by light activation of membrane-incorporated photosensitisers, facilitating intracellular drug release in the treatment of cancer. For PCI to be developed further, it is important to understand whether nerve damage is an impending side effect when treating cancers within or adjacent to nervous system tissue. Dorsal root ganglion (DRG) neurons and their associated satellite glia were subjected to PCI treatment in a 3D co-culture system following incubation with photosensitisers: meso-tetraphenylporphine (TPPS_2a_) or tetraphenylchlorin disulfonate (TPCS_2_a) and Bleomycin. Results from the use of 3D co-culture models demonstrate that a cancer cell line PCI30 and satellite glia were more sensitive to PCI than neurons and mixed glial cells, athough neurite length was affected. Neurons in culture survived PCI treatment under conditions sufficient to kill tumour cells, suggesting cancers within or adjacent to nervous system tissue could be treated with this novel technology.

## Graphical abstract

**Figure:**
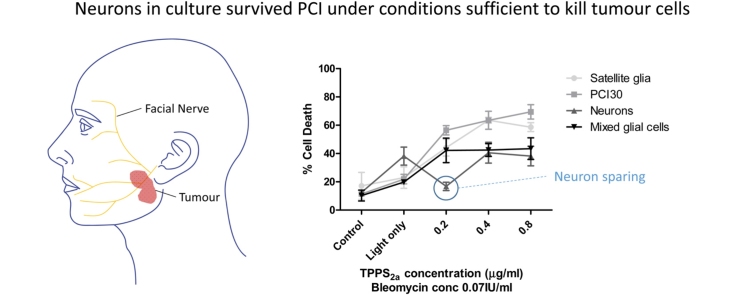

## 1 Introduction

Photochemical Internalisation (PCI) is a novel photochemical technology that facilitates the delivery of molecules into the cytosol of the cell. In PCI, a photosensitiser together with a therapeutic agent taken up via endocytosis. Exposure to light leads to endosomal rupture and the release of the therapeutic agent into the cytosol. The membrane rupture occurs through oxidative damage from ROS generated by the photosensitiser, but the light and photosensitiser doses used in PCI are too small to exert a lethal effect (in contrast to Photodynamic therapy (PDT), where the reactive oxygen species (ROS) damage induced by the photosensitiser is directly cytotoxic). There are a number of advantages for using PCI for drug delivery: it is site-specific and the enhanced delivery efficiency enables lower drugs doses to be applied using systemic administration (Selbo et al., 2002). By harnessing the improved delivery efficiency conferred by PCI, many of the unwanted side effects associated with using drugs with a molecular weight in excess of 1000 Da are reduced. For example it has been estimated that, once in the cytosol, as few as ∼500 Bleomycin molecules are sufficient to kill a cell (Poddevin et al., 1991).

PCI has potential to be an important new therapy for treatment of many cancers including head and neck cancer (HNC), bladder, and prostate. But for this treatment to be developed further, it is important to understand whether nerve damage is a potential side effect of PCI. To date many studies of PDT have described and confirmed nerve sparing even when the nerves are within the treatment field (Betz et al., 2007, DE Visscher et al., 2013, Green et al., 2013, Lou et al., 2004, Dole et al., 2005, Kubler et al., 2003). In experimental investigations to understand the clinical nerve sparing reported during certain PDT treatments, Wright et al., used 3D co-culture models to recreate the neuron-glial interactions and physical environment that influence neural cells and this approach enabled nerve-sparing photosensitisers to be distinguished from those that damage nerves (Wright et al., 2009). Importantly, a clinically-relevant mTHPC-mediated PDT treatment regime was identified which killed human breast cancer cells (MCF-7) in culture but spared neurons, reflecting the clinical observations associated with mTHPC (Wright et al., 2009). Subsequent studies using the same co-culture approach revealed the biochemical pathways likely to be responsible for the nerve sparing effect (Wright et al., 2012).

An efficient photosensitiser for PCI is meso-tetraphenylporphine (TPPS_2a_), which has two sulfonate groups on adjacent phthalate/phenyl rings. This amphiphilic structure with both a hydrophobic part and a hydrophilic part enables the photosensitiser to bind to the cell membrane and be taken up via adsorptive endocytosis (Friberg et al., 2003). A disadvantage of porphyrin photosensitisers is that they exhibit weak absorption at red/NIR wavelengths, which are used therapeutically since light in this wavelength range can penetrate more deeply into tissue. This led to the development of a chlorin based photosensitiser for PCI with two adjacent sulfonated groups which exhibits improved red wavelength absorption c. 650 nm, (TPCS_2a_, tetraphenylchlorin disulfonate). TPCS_2a_ has a low toxicity and high specificity towards endo/lysosomal membranes in tumour cells owing to its amphiphilic structure (Gaware et al., 2013). The clinically used formulation of this photosensitiser is known as Amphinex™.

Bleomycins are a family of water-soluble glycopeptidic antibiotics with at least four functional domains mediating DNA breakage, ion binding and O_2_ activation (Katare et al., 2014, Berg et al., 2011). Enhanced efficacy of PCI with Bleomycin has been documented in several preclinical studies and no severe side effects have been reported to date (Norum et al., 2009, Berg et al., 2010). Although Cisplatin, Carboplatin, Docetaxel and Gemcitabine are more commonly used in HNC therapy, their suitability for PCI is not fully established and some of these agents by themselves are reported to cause neurotoxicity (Mcwhinney et al., 2009, Park et al., 2013). A phase 1 clinical trial focused on treatment of head and neck cancer illustrated that TPCS_2a_-mediated photochemical internalisation of Bleomycin is safe and tolerable and further identified treatment doses (Sultan et al., 2016).

The effect of low dose TPCS_2a_/TPPS_2a_ PDT as well as the direct release of Bleomycin by PCI to nervous system tissue is unknown. Neurons have highly adapted systems for the axoplasmic transport of vesicles over greater distances than other cells, endo/exocytosis is coupled to electrochemical activity at synapses, and there is growing evidence for exosome movement between neurons and glia (Fang et al., 2015). These features, combined with the ability of neurons to resist ROS-mediated damage (Wright et al., 2012), make the effect of PCI on nerve cells hard to predict. The aim of this study was to investigate the effects of PCI agents on nervous system cells in 3D co-cultures. In line with the phase 1 clinical trial that used PCI to treat head and neck cancer, a cancer cell line representative of a head and neck squamous cell carcinoma (PCI30) was chosen for study alongside neural cells. Cells were organised within collagen hydrogels and treated with combinations of photosensitisers, chemotherapeutic agents and light. The sensitivity of neurons and glia to PCI treatments was investigated and compared to the PCI30 cell line in order to identify approaches that minimise nerve toxicity.

## 2 Materials and methods

### 2.1 Cell cultures

#### 2.1.1 Mixed glial cells

All experiments were performed according to the UK Animals (Scientific Procedures) Act (1986) and approved by the appropriate institutional ethical committee. Cortical mixed glial cells were isolated from cerebral cortices of postnatal, 2 day old (P2), Sprague Dawley rats, as adapted (Mccarthy and Devellis, 1980) and by (Chen et al., 2007). The cortices were finely chopped, placed in digest solution (0.2 mg/ml DNase, 250 μg/ ml trypsin in HBSS with Ca^2+^ and Mg^2+^ and 1% penicillin/streptomycin (P/S)) for 15 minutes at 37 °C. Subsequently, 10 ml DMEM containing 20% fetal calf serum (FCS) and 1% P/S (DMEM20S) was added and the cell suspension centrifuged for 5 minutes at 100 x g. The tissue pellet was resuspended in 20 ml DMEM20S and triturated with a 10 ml pipette followed by a Pasteur pipette, then a fire-polished Pasteur pipette. Following trituration, the suspension was left to settle on ice then the cell supernatant was passed through a 70 μm cell strainer and the top fraction containing dissociated cells collected. This process was repeated once more and cells were seeded into T75 flasks coated with 20 *μ*g/ml poly-D-lysine (PDL) at a ratio of 1 brain per flask in DMEM20S. Flasks were placed in a humidified incubator at 37 °C with 5% CO_2_, 95% air, with a media change weekly and cells were cultured for 2 weeks before use.

#### 2.1.2 DRG Neurons/Satellite glia

Neurons and satellite glial cells were cultured from dorsal root ganglia (DRGs), twenty of which were isolated from each freshly culled 250–350 g Sprague Dawley rat as previously described (Phillips et al., 2005). Using microscopic dissection, DRGs were removed, cleaned of all nerve roots and incubated for 90 min at 37 °C in Dulbecco's Modified Eagle's Medium (DMEM) containing 0.125% collagenase (Sigma), then triturated to produce a cell suspension. Culture medium (DMEM supplemented with 10% FCS and 1% P/S) was added and cells were separated from the collagenase by centrifugation for 5 min at 100 x g and resuspended in an appropriate volume of culture medium.

#### 2.1.3 Cell Lines

The tumour cells used in this study were the head and neck squamous cell carcinoma cell line, PCI30 (Lechner et al., 2013)(Generous gift from Dr Tim Fenton). PCI30 cells were cultured in T75 flasks in fully supplemented DMEM until 95% confluent whereupon they were removed using trypsinisation (0.25% trypsin–EDTA, 5 min, 37 °C). Cells were washed by centrifugation for 5 min at 100 x g and resuspended in an appropriate volume of culture medium.

##### 2.1.3.1 Cell culture models

Monolayer and three-dimensional (3D) cell culture models were used in this study. Analysis of photosensitiser uptake, co-localisation and assessment of neurite length were conducted using cells in monolayer cultures, whereas cell death assays used 3D cultures in order to trap non-adherent cells that would have been lost from monolayer cultures following treatment (Wright et al., 2009).

For monolayer cell culture, neural cells and PCI30 cells were grown on glass coverslips coated with 20 *μ*g/ml PDL. Cells were seeded in 100 *μ*l of culture medium (3.5 × 10^4^ cells/ml), allowed to adhere for 30 min, then incubated in 1 ml culture medium in 12-well culture plates.

For 3D cultures, gels were prepared using 80% v/v Type I rat tail collagen (2 mg/ml in 0.6% acetic acid; First Link) mixed with 10% v/v 10 × minimum essential medium (Sigma) and the mixture neutralised using 5.8% v/v neutralising solution (TAP Biosystems) before addition to 4.2% v/v cell suspension. 200 μl of the mixture, each containing 5 × 10^4^ of either DRG neurons/Satellite glia, mixed glial cells or PCI30 cells was added to individual wells of a 24-well plate and the gels were allowed to set at 37 °C for 10 min. Cellular gels were then immersed in 500 μl culture medium and maintained in culture at 37 °C, 5% CO_2_, for 4 days (DRG cells/Satellite glia) or 1 day (PCI30 and mixed glial cells) before experimentation; the 1-day option was chosen here to minimise time for proliferation of these rapidly dividing cells, whilst 4 days allowed neurite extension of DRG neurons.

### 2.2 Photochemical Internalisation

Culture medium was removed from coverslips or gels and cultures were incubated with photosensitiser drugs; TPCS_2a_ or TPPS_2a_ (based on previous *in vitro* studies with similar photosensitisers (Wang et al., 2013, Berg et al., 2010)) and/or the chemotherapeutic drug Bleomycin diluted in culture medium for 18 hrs in the dark at 37 °C, 5% CO_2_. The medium containing the drug compounds was removed, and the cultures washed with phosphate-buffered saline (PBS) before addition of fresh culture medium, and cultures were then incubated for a further 4 hrs. Cultures were then exposed to LED light with a peak wavelength of 420 nm and a fluence rate of 1.5 mW/cm^2^ (Thorlabs; 1000 mA, 750 mW) for 5 min, giving a total light dose of 0.6 J/cm^2^.The minimal thickness (<1 mm), relatively low cell density and transparent nature of the collagen gels ensured that all cells within them received an equivalent light dose (Wright et al., 2009). Controls were included in each experiment that involved excluding either the drug or the light (by wrapping the plate in foil during the illumination step) or both. Following incubation, gels and coverslips were incubated at 37 °C, 5% CO_2_ for a further 24 hrs unless otherwise stated.

#### 2.2.1 Fluorescence spectroscopy

PCI30 and neural cells were seeded into 96-well plates at 2–2.5 × 10^4^ cells per well overnight and then incubated with either TPCS_2a_ or TPPS_2a_ at different concentrations for 18 hrs. Thrice washing of cells and a further 4 hrs incubation with fresh culture medium was then carried out. The medium was replaced with clear medium (DMEM without phenol red or serum) for fluorescence measurements with excitation at 420 nm and detection at 650 nm using a LS50 B fluorescence spectrometer (Perkin Elmer) equipped with a 96-well plate reader and mean intracellular fluorescence for each photosensitiser calculated. Fluorescence from control cells without exposure to the photosensitiser was negligible.

### 2.3 Immunocytochemistry

Following fixation, cell permeabilisation was performed using 0.5% TritonX-100 (Sigma) for 30 min. Following 3 × 5 min washes, non-specific binding was blocked with 5% normal goat serum (Dako) in PBS for 30 min. After another wash step, primary antibodies were diluted 1:400 in PBS (mouse anti-β III-tubulin; Sigma) and incubated overnight at 4 °C. Following 3 × 10 min washes, secondary antibody, anti-mouse IgG DyLight 488 (Vector Laboratories) was diluted 1:300 in PBS and added for 90 min. Hoechst 33258 (1 μg/ml) was also added into the secondary antibody incubation to stain nuclei. Omission of a primary or secondary antibody was routinely used as a control. Incubation times for coverslips were half that for gels except for an overnight incubation in primary antibodies. Gels and coverslips were stored in PBS at 4 °C.

### 2.4 Cell death assay

Cell death was assessed using propidium iodide (PI; Sigma) staining in combination with Hoechst 33258. Briefly, PI was added to cultures at 200 μg/ml in cell culture medium and left to incubate for 15 min at 37 °C. The medium was then removed and the cultures were rinsed in PBS before fixing in 4% paraformaldehyde (PFA) at 4 °C. Gels were incubated with Hoechst 33258 (1 μg/ml; Sigma) in PBS for 10 min, before 3 × 5 min washes in PBS. Fluorescence microscopy was used to determine cell viability. Images were captured using a Zeiss Axiolab A1 fluoroscence microscope and Zeiss AxioCam C1. Three fields were randomly selected per gel. The % of dead cells for each cell population was determined by counting the number of PI stained cells and the total number of cells, as determined by Hoechst staining. For neurons, the number of βIII-tubulin immunopositive cells was calculated as a percentage of the total number of cells/field and compared to the number of PI stained cells to determine cell death.

### 2.5 Image analysis and quantification

Neurite length was determined from images captured using the fluorescence microscope. The length of each neurite captured per image was measured by manual tracing using ImageJ.

Confocal microscopy (Zeiss LSM 710) was used to capture images for analysis of co-localisation. LysoTracker^®^ Green DND-26 (ThermoFisher Scientific) was used to label lysosomes and their localisation relative to the photosensitiser was determined. Colocalization analysis was performed on single-plane confocal images (3 images per coverslip) using Volocity™ 6.4 (Perkin Elmer) software which calculated the Pearson’s correlation coefficient and the overlap coefficient. Pearson's correlation measures the strength of the association between the two fluorescents giving values of between +1 and −1, where +1 suggests a total positive correlation, 0 is no correlation and −1 a total negative correlation. Similarly, the overlap coefficient measures co-localisation of fluorescent signals to generate values between 0-1, with 0 being no overlap and 1 perfect image registration.

### 2.6 Statistical analysis

Normality tests were performed on all data to determine which test was appropriate and one-way ANOVA or t-tests were performed if data followed a normal distribution. A one-way ANOVA was followed by a Dunnett’s post hoc test to compare multiple conditions against the control, or Tukey’s multiple comparisons test to compare groups. When comparing the mean differences between groups that have been split on two independent variables a two-way Anova was performed. For all tests, *p < 0.05, **p < 0.01, ***p < 0.001 and ****p < 0.0001 were considered to be significant.

## 3 Results

### 3.1 Increasing uptake of photosensitisers TPCS_2a_ and TPPS_2a_ with increasing concentrations by neural cells

**Fig. 1:**
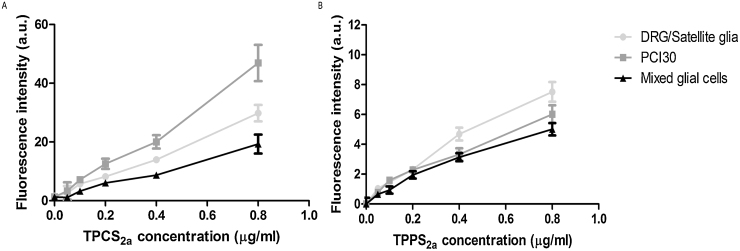
Uptake of TPCS_2a_ and TPPS_2a_ by neural and PCI30 cells. Fluorescence intensity at 660 nm of 0.05–0.8 μg/ml (A) TPCS_2a_ and (B) TPPS_2a_ taken up by PCI30, mixed glial cells and DRG neurons/Satellite glia following 18 hrs incubation with an excitation wavelength of 420 nm. Mean ± SEM, n = 3.

PCI30 and neural cells were incubated with TPCS_2a_ and TPPS_2a_ using a range of concentrations from 0.05 − 0.8 μg/ml for 18 hrs following which the mean intracellular fluorescence for each photosensitiser was assessed. For each cell type, the fluorescence intensity for TPCS_2a_ at concentrations of 0.2–0.8 μg/ml was much greater when compared to that of TPPS_2a_ (Fig. 1A and B). Additionally, PCI30 cells exhibited considerably greater fluorescence intensity than neural cells when incubated with higher concentrations of TPCS_2a_, suggesting greater uptake of the photosensitiser (see Fig. 1A).

### 3.2 Photosensitisers TPCS_2a_ and TPPS_2a_ localise within endosomes in neural cells

**Fig. 2:**
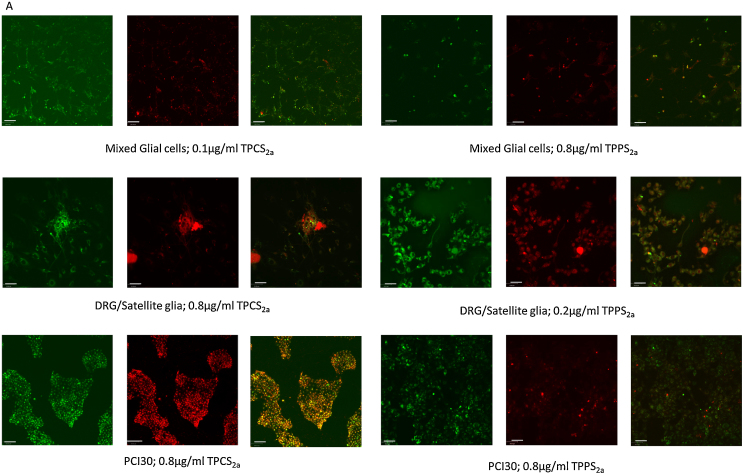

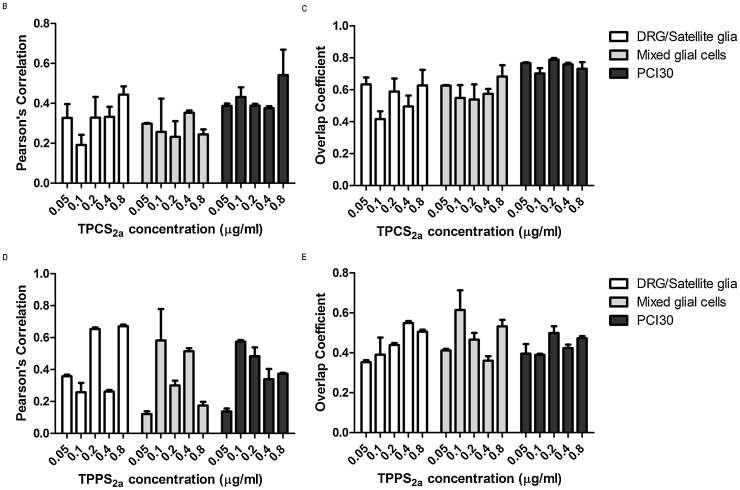
Photosensitisers localise to lysosomes within cells. Confocal micrographs exhibit co-localisation of lysotracker (green) and photosensitisers (red), TPCS_2a_ and TPPS_2a_ within neural cells. Scale bar = 35 μm. (B) and (D) Pearson’s correlation coefficient, (C) and (E)overlap coefficient illustrating the overlap photosensitiser and lysotracker used as a marker for endosomes. Mean ± SEM, n = 3.

Confocal micrographs confirm that both TPCS_2a_ and TPPS_2a_ localised within endosomes intracellularly, as seen in Fig. 2. Photosensitiser colocalisation with lysotracker was analysed via quantitative analysis of confocal images. Two different values were calculated; the Pearson’s correlation coefficient and the overlap coefficient (Dunn et al., 2011). For TPCS_2a_, both Pearson’s correlation coefficient and overlap coefficient values were higher in PCI30 cells at all concentrations compared to mixed glial cells and DRG/satellite glia, suggesting a greater proportion of photosensitiser could be located within the endosomes of the cancer cell line. This same was not true for TPPS_2a_ with no definitive pattern observed for both Pearson’s correlation coefficient and overlap coefficient values.

### 3.3 Neural cells display limited dark toxicity in the presence of TPCS_2a_ and TPPS_2a_

**Fig. 3:**
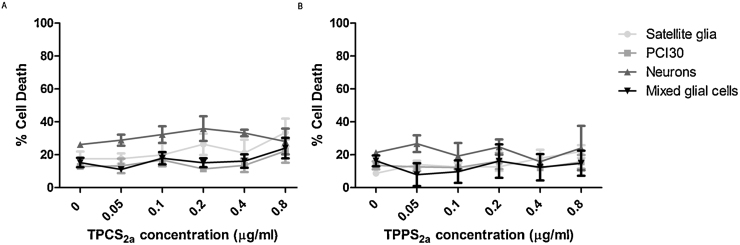
Dark toxicity of TPCS_2a_ and TPPS_2a._ PCI30, primary mixed glial cells, neurons and satellite glia were incubated with 0.5–0.8 μg/ml (A) TPCS_2a_ or (B) TPPS_2a_ for 18 hrs, which showed to have minimal effect upon cell viability. Mean ± SEM, n = 6.

To establish whether the presence of photosensitisers in culture have a toxic effect upon neurons and mixed glial cells in the absence of light, PCI30 and neural cells were incubated with TPCS_2a_ and TPPS_2a_ using a range of concentrations from 0.05–0.8 μg/ml for 18 hrs. Live/dead assays established the dark toxicity of the photosensitisers which is minimal for all cells types with neither neural cells nor the cancer cell line showing significant cell death in the presence of the compounds, as seen in Fig. 3.

### 3.4 Phototoxicity of TPCS_2a_ and TPPS_2a_

**Fig. 4:**
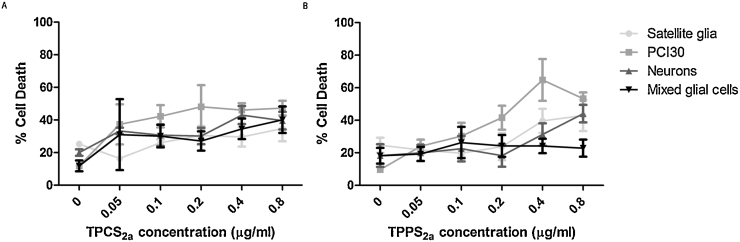
Phototoxicty of TPCS_2a_ and TPPS_2a._ PCI30, primary mixed glial cells, neurons and satellite glia were incubated with 0.05–0.8 μg/ml TPCS_2a_ or TPPS_2a_ for 18 hrs. Cells were irradiated 0.6 J/cm^2^ using an LED light source with a wavelength of 420 nm. Results are shown as mean percentage cell death against increasing concentrations of photosensitiser. Mean ± SEM, n = 6.

The phototoxicity induced by both TPCS_2a_ and TPPS_2a_ was investigated at a range of concentrations under a light exposure of 0.6 J/cm^2^. Dose dependent cell death was found with both photosensitisers and it was noted that higher concentrations of TPPS_2a_ (0.4–0.8 μg/ml) resulted in higher cell death when compared to equivalent concentration of TPCS_2a_ (Fig. 4). From this information, a starting concentration of 0.2 μg/ml was chosen for subsequent PCI experiments due to these concentrations achieving approximately 50% cell death (PDT_50_) of PCI30 cells. Determination of treatment conditions corresponding to the PDT_50_ is important when selecting the optimum treatment parameters for PCI where a sub-lethal PDT effect is the aim.

### 3.5 Increasing concentrations of Bleomycin results in increased cell death

**Fig. 5:**
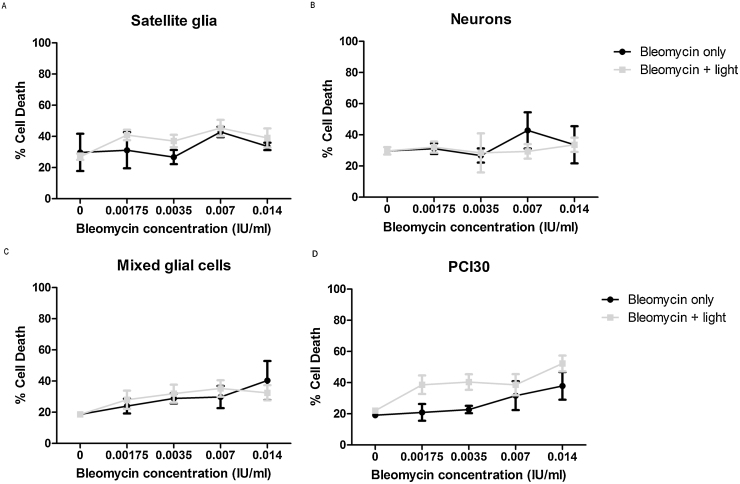
Cytotoxicity of Bleomycin with or without light exposure. DRG neurons, satellite glia, PCI30, and mixed glial cells were incubated with 0.00175–0.014 IU/ml of Bleomycin for 18 hrs. Cells were illuminated with 0.6 J/cm^2^ using a LED light source with a wavelength of 420 nm and t-test results revealed no significant increases in cell death due to illumination. Mean ± SEM, n = 6.

The cytotoxicity induced by the anti-cancer drug Bleomycin with and without light was examined prior to the combination with PDT treatment. In control gels without any applied drug but with light exposure, no change in viability was observed. Bleomycin produced similar toxicity in neurons, satellite glia, mixed glial cells and neurons alone and the presence of the light did not increase death significantly, as revealed by t-tests. PCI30 cell death did however appear to increase in the presence of light, albeit not significantly as examined by a t-test (Fig. 5).

### 3.6 Cytotoxicity of TPCS_2a_ PCI combining Bleomycin

**Fig. 6:**
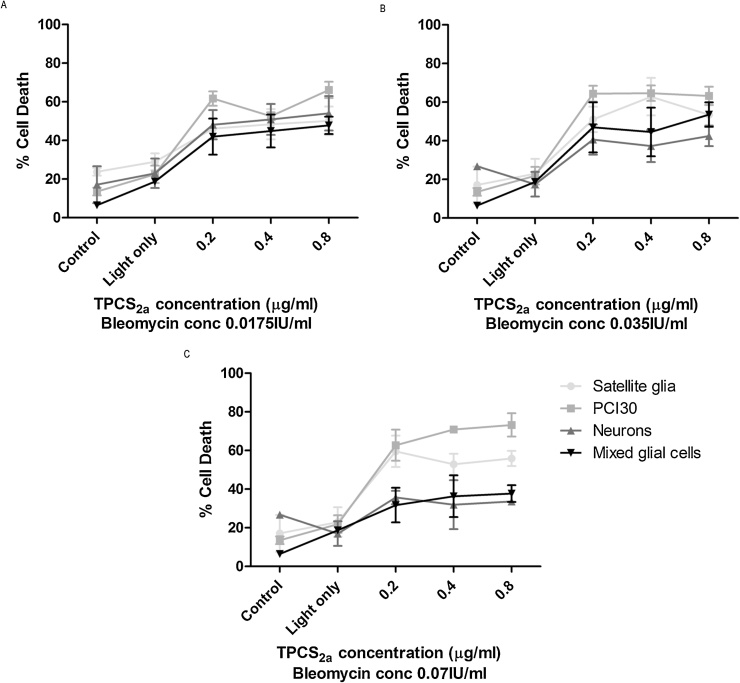
Sensitivity of cells to TPCS_2a_ PCI of Bleomycin. PCI30, mixed glial cells, neurons and satellite glia incubated with 0.2–0.8 μg/ml TPCS_2a_ and 0.00175–0.07 IU/ml of Bleomycin for 18 hrs before irradiation using a LED light source. Two-way ANOVA revealed a main effect of cell type, as well as concentration of TPCS_2a_ in increased cell death and an interaction between cell type and TPCS_2a_ concentration. Mean ± SEM, n = 6.

The effect of using PCI to release Bleomycin was investigated using TPCS_2a_ with approximately the PDT_50_ doses (0.2, 0.4 and 0.8 μg/ml (Fig. 4A)). All gels were incubated with drugs for 18 hrs and light exposures of 5 mins were employed for each case to give a total light dose of 0.6 J/cm^2^. For all concentrations, neuronal cell death was significantly lower than that of the PCI30 cell line, suggesting neurons are more resistant to this treatment than PCI30 cells. Mixed glial cells also exhibited less cell death than the cancer cell line. Satellite glia displayed a high percent of cell death following treatment, particularly in the presence of higher concentrations of Bleomycin, indicating that the satellite glia were more susceptible to PCI treatment than neurons and mixed glial cells (Fig. 6).

### 3.7 Cytotoxicity of TPPS_2a_ PCI combining Bleomycin

The PCI effect combining Bleomycin in primary neural cells and PCI30 cells was investigated using TPPS_2a_ with approximately the PDT_50_ doses. All gels were incubated with drugs for 18 hrs and light exposures of 5 mins were employed for each case. Similar to results obtained for TPCS_2a_ neuronal cell death was significantly lower than that of the PCI30 cell line for all concentrations of both TPPS_2a_ and Bleomycin. Satellite glia however exhibited similar levels of cell death as PCI30 cells, much higher than neurons or mixed glial. Mixed glial cells appear to exhibit greater cell death than neurons in the presence of TPPS_2a_.

### 3.8 No increased neuronal death 48 and 72 hours post treatment with TPCS_2a_ PCI of Bleomycin

**Fig. 7:**
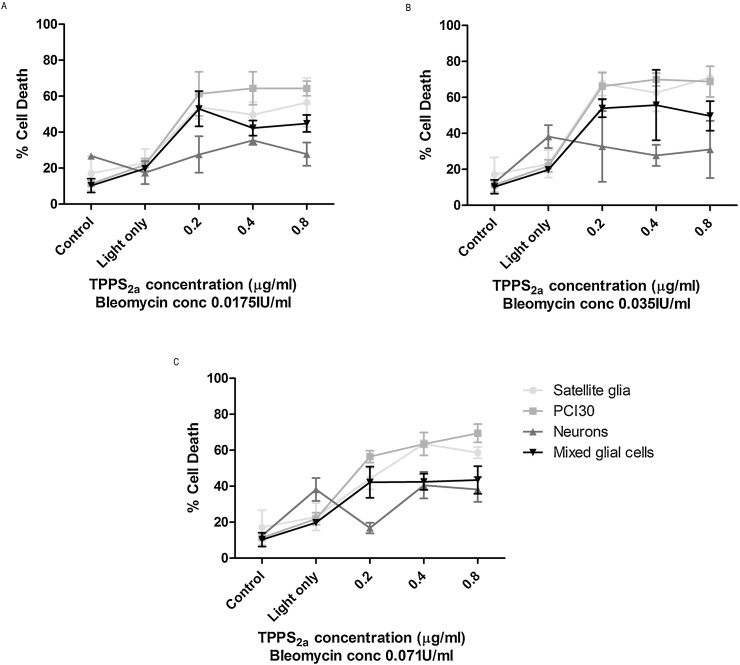
Sensitivity of cells to TPPS_2a_ PCI of Bleomycin. PCI30, mixed glial cells neurons and satellite glia incubated with 0.2–0.8 μg/ml TPPS_2a_ and 0.00175–0.07IU/ml of Bleomycin for 18 hrs before irradiation using a LED light source. Two-way ANOVA revealed a main effect of cell type, as well as concentration of TPCS_2a_ in increased cell death and an interaction between cell type and TPCS_2a_ concentration. Mean ± SEM, n = 6.

**Fig. 8:**
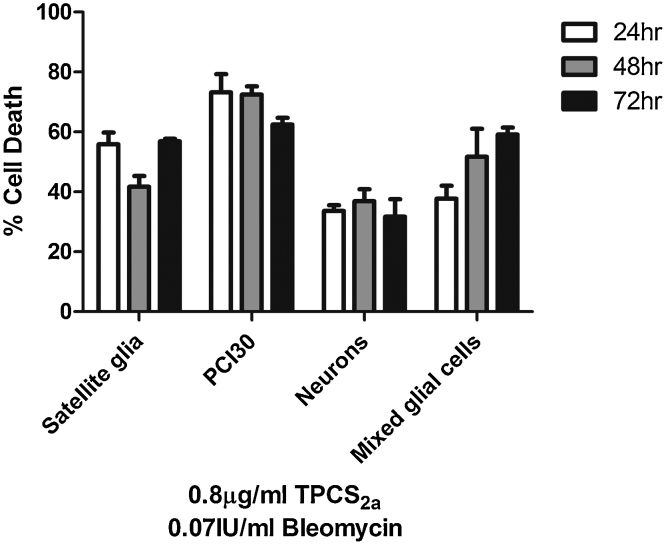
Cell death 48 and 72 hours post PCI treatment with TPCS_2a_ and Bleomycin. PCI30, mixed glial cells, neurons and satellite glia incubated with 0.8 μg/ml TPCS_2a_ and 0.07IU/ml of Bleomycin for 18 hrs before irradiation using a LED light source. Cell death was assessed after 24, 48 and 72 hours and one-way ANOVA revealed no significant increase in cell death over time. Mean ± SEM, n = 6.

**Fig. 9:**
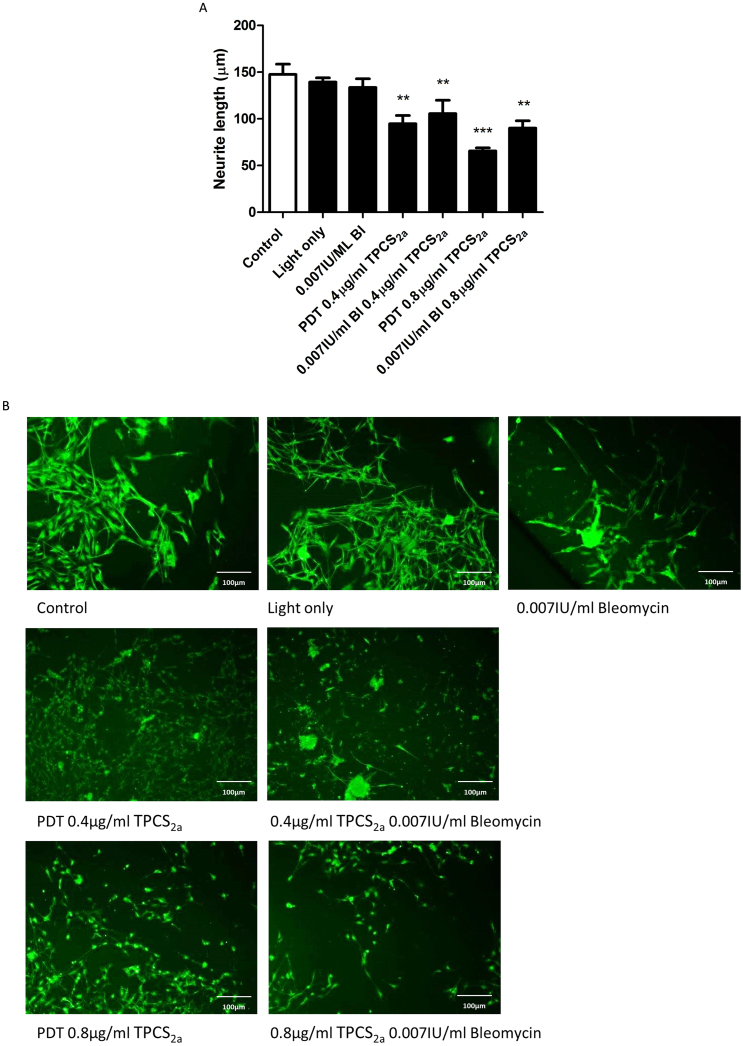
Neurite length significantly decreased following PDT and PCI treatment. (A) Neurite length of primary DRG neurons was significantly reduced followed PDT treatment using 0.4 and 0.8 μg/ml TPCS_2a_ and PCI treatment in combination with 0.07IU/ml of Bleomycin (Bl) when compared to control. Mean ± SEM, n = 6, One-way ANOVA with Dunnett’s post hoc test, **p < 0.01, ***p < 0.001. (B) Fluorescence micrographs exhibit differences in neurons stained for β-III tubulin (green) post treatment.

The PCI effect over time in combination with 0.07 IU/ml Bleomycin in primary neural cells and the PCI30 cancer cell line was investigated using 0.8 μg/ml TPCS_2a_. Similar to previous experiments, all gels were incubated with drugs for 18 hrs and light exposures of 5 mins were employed for each case. Results reveal that neuronal death did not increase over time, and similar results were seen with satellite glia. No increase in cell death overtime of PCI30 cells was seen, but instead a small but non-significant decrease was seen, presumably due to cell proliferation. Interesting, mixed glial cell death appeared to increase over time (Fig. 7, Fig. 8, Fig. 9).

### 3.9 Treatment of TPCS_2a_ PDT and PCI combining Bleomycin reduced neurite length

Neurite length was assessed following treatment with PDT and PCI combining 0.07 IU/ml Bloemycin with 0.4 and 0.8 μg/ml TPCS_2a_. And as before, light exposures of 5 mins were employed and incubation with drugs for 18 hrs. Results reveal that both PDT and PCI significant reduced neurite length when compared to control conditions. Irradiation with light and incubation with 0.007 IU/ml Bleomycin alone however, did not affect neurite length.

## 4 Discussion

The overall aim of the project was to understand the effects of PCI treatment on mammalian peripheral nerve cells in order to minimise nerve toxicity in future clinical applications. This is the first study to investigate the effects of PCI upon nervous system cells, which is an important consideration for developing PCI to treat tumours adjacent to or within nervous system tissue. One of the great therapeutic advantages that PDT has over surgery is preservation of nerve function as the other main therapy for head and neck cancer causes demyelination especially with irradiation. We have shown in previous clinical studies that nerve function remains intact after PDT treatment and this has been confirmed using *in vitro* models. Early indications are that nerve sparing is also seen with PCI and this experimental study carried out under controlled conditions indicates this is indeed the case.

Using advanced cell culture models, the photosensitisers TPCS_2a_ and TPPS_2a_ were investigated along with the chemotherapeutic drug Bleomycin. Cells included neurons and glial cells from the peripheral and central nervous system and controls used a relevant head and neck cancer cell line, PCI30. These cell culture models provide a powerful system in which to study neural cell responses to PCI treatment as they can be tightly controlled and easily monitored and are usually less expensive and less time consuming than animal models (Griffith and Swartz, 2006). The interactions between neurons and glial cells are important in determining the phenotype and survival of each and the 3D environment allows neurons and glia to interact in a manner that mimics their interdependence in neural tissue.

The key findings of this study is that DRG neurons are less sensitive to TPCS_2a_ and TPPS_2a_-mediated PCI than their associated satellite glia and the tumour cell line PCI30, both of which demonstrated a dose-dependent increase in cell death over a range of photosensitiser and chemotherapeutic drug concentrations. CNS glia (mixed glia) consisting of astrocyte, oligodendrocytes and microglia, also demonstrated a dose-dependent increase in cell death. These results suggest that DRG neurons are able to survive PCI treatment with the chosen photosensitisers (TPCS_2a_ and TPPS_2a_) and Bleomycin treatments that kill tumour cells.

Firstly uptake of both TPCS_2a_ and TPPS_2a_ in neurons and satellite glial, cancer cells and mixed glial cells was characterised revealing that all cells were capable of taking up both photosensitisers and that the cancer cell line exhibited greater uptake of TPCS_2a_ when compared with the primary cells. Comparing the photosensitiser fluorescence levels in each cell type, TPCS_2a_ yielded higher fluorescence in all cell types compared to TPPS_2a_ at the same doses. This is in line with studies that have also examined uptake of both photosensitisers (Martinez De Pinillos Bayona et al., 2017). However since TPCS_2a_ is a more efficient fluorophore than TPPS_2a_, the higher intracellular fluorescence does not imply that higher concentrations of TPCS_2a_ are taken up by cells (Lilletvedt et al., 2010). Immunostaining confirmed that the photosensitisers were colocalised with Lysotracker Green, which is consistent with endolysosomal localisation of the photosensitiser. Additionally it was confirmed that the presence of the photosensitisers in the absence of light had no toxic effects upon cells. Phototoxicity of TPCS_2a_ and TPPS_2a_ was assessed, which showed that the PCI30 cell line was more susceptible to TPPS_2a_ than primary neural cells, whilst this pattern was not observed for TPCS_2a._From these data, the sub-lethal phototoxic threshold (PDT_50_ i.e. 50% cell death) of light and photosensitiser (PDT) was established for subsequent studies.

Bleomycin cytotoxicity is caused by single and double-stranded DNA damage and at high doses can result in pneumonitis and subsequent lung fibrosis (Berg et al., 2005). PCI reduces the Bleomycin dose required, and Berg et al., performed PCI using *in vitro* and *in vivo* human sarcoma, human colorectal adenocarcinoma and murine colon carcinoma models using TPPS_2a_ (0.7 μg mL^−1^) and Bleomycin (0.14 IU mL^−1^). The study demonstrated that use of Bleomycin in combination with PCI significantly enhanced cytotoxicity by a factor of three compared to Bleomycin alone *in vitro* (Berg et al., 2005). The *in vitro* studies here demonstrated that Bleomycin alone caused cell death of DRG and satellite glial cells, PCI30 cells and mixed glial cells and illumination of gels following administration of Bleomycin did not increase death significantly.

PCI studies combining Bleomycin with TPPS_2a_ demonstrated a dose dependent increase in cell death following 18 hours incubation with both photosensitiser and Bleomycin. Importantly the cancer cell line was more susceptible to PCI than neurons, with significant differences observed between PCI30 and DRG neurons, and at the highest concentrations of TPPS_2a_ and Bleomycin used in this study, twice the amount of PCI30 cells death than neurons. These results suggest that PCI treatment combining TPPS_2a_ and Bleomycin has the potential to spare neurons whilst killing cancer cells. Similarly, PCI studies combining Bleomycin with TPCS_2a_ demonstrated a dose dependent significant increase in cell death following 18 hours incubation with both photosensitiser and Bleomycin. At higher concentrations of Bleomycin (0.07 IU/ml), a significant difference was observed between PCI30 cells and DRG neurons, with reduced cell death observed in DRG neurons suggesting TPCS_2a_ and Bleomycin spare neurons whilst killing cancer cells. However, it is important to note that as the photosensitiser in PCI is located in close proximity to the molecule to be internalised, photochemical treatment could potentially damage not only the endosomal membrane, but also the molecule to be internalised (Hogset et al., 2004). There have been several studies that have indicated that endocytosed molecules may be damaged by the PCI procedure (Berg et al., 1999, Prasmickaite et al., 2000). This may explain why the highest concentrations of photosensitiser do not results in increased cell death in PCI studies.

TPCS_2a_ has been proposed as an optimal photosensitiser for clinical PCI, owing to suitable photophysical and photobiological properties (Wang et al., 2013, Berg et al., 2011) and more recently, a phase 1 clinical trial has found that administration of TPCS_2a_ is safe and tolerable to human patients and identified 0.25 mg/kg as the recommended treatment dose (Sultan et al., 2016). TPPS_2a_ has been investigated *in vitro* with promising results, however is less suitable for clinical development since its red absorption is comparatively weak (Martinez De Pinillos Bayona et al., 2016). As a result, further studies were undertaken in which PCI using TPCS_2a_ and Bleomycin was investigated in more detail.

The potential nerve-sparing effects observed *in vitro* 24 h after PCI treatment were investigated further by extending the time between treatment and cell death analysis; as bleomycin induces single and double-strand DNA damage cell death can occur at later timepoints. There was no increase in neuron death detected at 48 h and 72 h, confirming that neurons survived PCI treatment over a longer term. Importantly, despite the survival of DRG neurons, PCI treatment caused as a substantial reduction in neurite length. Wright et al. demonstrated a similar phenomenon; sparing of neurons but a reduction in neurite length following mTHPC-mediated PDT treatment (Wright et al., 2009). Since both that PDT study and this PCI study used neurons in co-culture with glial cells, which naturally provide trophic support to neurons, it is possible that the reduction in neurite length was a consequence of glial cell depletion rather than a direct effect of treatment on neurons. It will be important to explore this further, since neurite damage without neuronal death might provide an opportunity for any short-term loss of function to be reversed.

PCI using Bleomycin has also been investigated for brain cancer. *In vivo* studies showed improved survival of animals bearing the F98 glioma model using a combination of Bleomycin PCI and an epsilon prototoxin *versus* controls (Hirschberg et al., 2009). However, whether there was an effects of PCI upon the surrounding cellular environment, specific possible effects to astrocytes, oligodendrocytes and microglia, was not described. Here we report that mixed glial cells are similar to neurons; less sensitive to PCI treatment than a cancer cell line, however, an increase in cell death overtime i.e. 48 and 72 hours post PCI treatment was noted.

This study indicates that PCI treatment combining Bleomycin in combination with either TPCS_2a_ or TPPS_2a_ allows neurons to survive but kills cancer cells and satellite glia. This offers the potential for developing clinical PCI treatments for cancers within or adjacent to sensitive nervous system tissue, e.g. cancers of the head and neck, bladder, prostate and bone metastases in the spine. The 3D co-culture models used here provided a useful insight into the comparative sensitivity of different cell types to PCI. In an era of organ preserving cancer therapy, PDT and PCI has shown *in vitro* and *in vivo* to be function preserving therapies, especially in head and neck cancer, where surgery has an adverse effect on functions such as speech and swallowing.
