# Supplementary material for: Could clinical photochemical internalisation be optimised to avoid neuronal toxicity?
Source: Int J Pharm. 2017 Aug 7;528(1-2):133–43. doi: 10.1016/j.ijpharm.2017.05.071 (PMC5571751; doi:10.1016/j.ijpharm.2017.05.071)
Supplement: Supplementary file 1 [file mmc1.docx]

Supplementary Data

**Section 3.1**

Figure 1 (a)

Analysis of variance by two-way ANOVA revealed a main effect of TPCS_2a_ concentration (p < 0.0001), as well as the presence of cell type (p < 0.0001), and an interaction between both p < 0.0001).

Figure 1 (b)

Analysis of variance by two-way ANOVA revealed a main effect of TPPS_2a_ concentration (p < 0.0001), as well as cell type (p < 0.0001), and an interaction between both (p < 0.01).

In addition, statistical analysis revealed that there was a greater uptake of all TPCS_2a_ than TPPS_2a_ at concentrations of 0.2, 0.4 and 0.8µg/ml in all cell types.

**Section 3.3**

Figure 3 (a)

One-way ANOVA revealed significant differences in cell death between cell types at specific concentrations of TPCS_2a_.

| Mixed glia vs Neurons @0.05µg/ml | | | | * |
| --- | --- | --- | --- | --- |
| Mixed glia vs Neurons @0.2µg/ml | | | | * |
| PCI30 vs Neurons @0.2µg/ml | | |  | * |
|  |  |  |  |  |

Figure 3 (b)

One-way ANOVA revealed significant differences in cell death between cell types at 0.05µg/ml TPCS_2a_.

| PCI30 vs Neurons @0.05µg/ml |  | * |
| --- | --- | --- |
| Mixed glia vs Neurons @0.05µg/ml | | * |

**Section 3.4**

Figure 4 (a)

Analysis of variance by two-way ANOVA revealed a significant main effect of TPCS_2a_ concentration (p < 0.01), as well as the presence of cell type (p < 0.05).

| PCI30 vs Neurons control |  | * |
| --- | --- | --- |
| Neurons vs Mixed glia control |  | * |
| Satellite glia vs Mixed glia control | | ** |
| Satellite glia vs PCI30 control |  | ** |

In addition, statistical analysis revealed a significant increase in cell death at TPCS_2a_ concentration of 0.8µg/ml when compared to control.

|  | **TPCS_2A_ (µg/ml) Mixed glia** | | | | | |
| --- | --- | --- | --- | --- | --- | --- |
|  | **0** | **0.05** | **0.1** | **0.2** | **0.4** | **0.8** |
| **0** | ns | ns | ns | ns | ns | * |
| **0.05** | ns | ns | ns | ns | ns | ns |
| **0.1** | ns | ns | ns | ns | ns | ns |
| **0.2** | ns | ns | ns | ns | ns | ns |
| **0.4** | ns | ns | ns | ns | ns | ns |
| **0.8** | ns | ns | ns | ns | ns | ns |

Figure 4 (b)

Analysis of variance by two-way ANOVA revealed a main effect of TPPS_2a_ concentration (p < 0.0001), as well as cell type (p < 0.01), and an interaction between both (p < 0.05).

Statistical analysis revealed significant differences in cell death at various TPPS_2a_ concentrations and between cell types.

| PCI30 vs Mixed glia @0.4µg/ml | | | | | * | |  |  |
| --- | --- | --- | --- | --- | --- | --- | --- | --- |
| PCI30 vs Mixed glia @0.8µg/ml | | | | | ** | |  |  |
|  | **TPPS_2A_ (µg/ml) PCI30 Cells** | | | | | | | |
|  | **0** | **0.05** | **0.1** | **0.2** | | **0.4** | | **0.8** |
| **0** | ns | ns | ns | ns | | *** | | ** |
| **0.05** | ns | ns | ns | ns | | * | | ns |
| **0.1** | ns | ns | ns | ns | | * | | ns |
| **0.2** | ns | ns | ns | ns | | ns | | ns |
| **0.4** | ns | ns | ns | ns | | ns | | ns |
| **0.8** | ns | ns | ns | ns | | ns | | ns |

**Section 3.5**

Figure 5 (c)

Analysis of variance by two-way ANOVA revealed a main effect of Bleomycin concentration upon mixed glial cells (p < 0.05), but the presence of absence of light does not have a significant effect.

Figure 5 (d)

Analysis of variance by two-way ANOVA revealed a main effect of Bleomycin concentration upon PCI30 cells (p < 0.0001), and presence of light also has a significant effect (p < 0.0001).

|  | **Bleomycin (IU/ml) PCI30 Cells** | | | | | |
| --- | --- | --- | --- | --- | --- | --- |
|  | **0** | **0.00175** | **0.0035** | **0.007** | **0.014** |  |
| **0** | ns | ns | ns | ** | *** |  |
| **0.00175** | ns | ns | ns | ** | *** |  |
| **0.0035** | ns | ns | ns | * | *** |  |
| **0.007** | ns | ns | ns | ns | ns |  |
| **0.014** | ns | ns | ns | ns | ns |  |

|  | **Bleomycin + Light (IU/ml) PCI30 Cells** | | | | | |
| --- | --- | --- | --- | --- | --- | --- |
|  | **0** | **0.00175** | **0.0035** | **0.007** | **0.014** |  |
| **0** | ns | ns | ns | * | * |  |
| **0.00175** | ns | ns | ns | ns | ns |  |
| **0.0035** | ns | ns | ns | ns | ns |  |
| **0.007** | ns | ns | ns | ns | ns |  |
| **0.014** | ns | ns | ns | ns | ns |  |

In addition, there was a significant increase in PCI30 cell death in the presence of light at concentrations of 0.00175 and 0.0035 IU/ml when compared to cells that were not treated with light.

**Section 3.6**

Figure 6 (a)

Two-way ANOVA revealed a main effect of cell type (p<0.0001), as well as concentration of TPCS_2a_ (p<0.0001) in increased cell death and an interaction between cell type and TPCS_2a_ concentration (p<0.0001).

ANOVA revealed significant differences in cell death between cell types at specific concentrations of photosensitiser

| Bleomycin 0.00175 IU/ml | |  |
| --- | --- | --- |
| Mixed glia vs Neurons @0.2µg/ml | | * |
| Satellite glia vs Neurons @0.2µg/ml | | *** |
| PCI30 vs Neurons @0.2µg/ml |  | *** |
| Mixed glia vs Neurons @0.4µg/ml | | * |
| Satellite glia vs Neurons @0.4µg/ml | | ** |
| PCI30 vs Neurons @0.4µg/ml |  | ** |
| Satellite glia vs Neurons @0.8µg/ml | | * |
| PCI30 vs Neurons @0.8µg/ml |  | *** |

Figure 6 (b)

Two-way ANOVA revealed a main effect of cell type (p<0.05), as well as concentration of TPCS_2a_ (p<0.01) in increased cell death and an interaction between cell type and TPCS_2a_ concentration (p<0.0001).

ANOVA revealed significant differences in cell death between cell types at specific concentrations of photosensitiser

| Bleomycin 0.0035 IU/ml |  |  |
| --- | --- | --- |
| PCI30 vs Neurons @0.8µg/ml | * |  |

Figure 6 (c)

Two-way ANOVA revealed a main effect of cell type (p<0.0001), as well as concentration of TPCS_2a_ (p<0.0001) in increased cell death and an interaction between cell type and TPCS_2a_ concentration (p<0.01).

ANOVA revealed significant differences in cell death between cell types at specific concentrations of photosensitiser

| Bleomycin 0.007 IU/ml |  |
| --- | --- |
| PCI30 vs Neurons @0.2µg/ml | *** |
| PCI30 vs Mixed Glial cells @0.2µg/ml | * |
| Neurons vs Satellite glia @0.2µg/ml | * |
| PCI30 vs Neurons @0.4µg/ml | * |
| PCI30 vs Mixed Glial cells @0.4µg/ml | * |

**Section 3.7**

Figure 7 (a)

Two-way ANOVA revealed a main effect of cell type (p<0.01), as well as concentration of TPCS_2a_ (p<0.0001) in increased cell death and an interaction between cell type and TPCS_2a_ concentration (p<0.05).

ANOVA revealed significant differences in cell death between cell types at specific concentrations of photosensitiser

| Bleomycin 0.00175 IU/ml | |  |
| --- | --- | --- |
| Mixed glia vs Neurons @0.2µg/ml | | * |
| PCI30 vs Neurons @0.2µg/ml | | ** |
| PCI30 vs Neurons @0.4µg/ml |  | * |
| Mixed glia vs Neurons @0.4µg/ml | | * |

Figure 7 (b)

Two-way ANOVA revealed a main effect of cell type (p<0.001), as well as concentration of TPCS_2a_ (p<0.0001) in increased cell death and an interaction between cell type and TPCS_2a_ concentration (p<0.05).

ANOVA revealed significant differences in cell death between cell types at specific concentrations of photosensitiser

| Bleomycin 0.0035 IU/ml |  |  |
| --- | --- | --- |
| PCI30 vs Neurons @0.2µg/ml | ** |  |

Figure 7 (c)

Two-way ANOVA revealed a main effect of cell type (p<0.0001), as well as concentration of TPCS_2a_ (p<0.0001) in increased cell death and an interaction between cell type and TPCS_2a_ concentration (p<0.01).

ANOVA revealed significant differences in cell death between cell types at specific concentrations of photosensitiser

| Bleomycin 0.007 IU/ml |  |
| --- | --- |
| PCI30 vs Neurons @0.2µg/ml | *** |
| PCI30 vs Mixed Glial cells @0.2µg/ml | ** |
| PCI30 vs Neurons @0.8µg/ml | *** |
| Neurons vs Satellite Glial cells @0.8µg/ml | * |
| Neurons vs Mixed Glial cells @0.8µg/ml | * |
